# Management of an Unusual Central Nervous System Metastasis With Linear Accelerator Radiosurgery in a Low-Middle Income Country

**DOI:** 10.7759/cureus.19806

**Published:** 2021-11-22

**Authors:** Martin Mosquera, Raul Puente-Vallejo, Jose E Leon-Rojas

**Affiliations:** 1 School of Medicine, Faculty of Health and Life Sciences, Universidad Internacional del Ecuador, Quito, ECU; 2 Medical Research Department, NeurALL Research Group, Quito, ECU; 3 Radiation Oncology Department, Hospital Solon Espinosa Ayala (SOLCA), Quito, ECU

**Keywords:** radiosurgery, linear particle accelerator, case report, brain metastasis, neuroendocrine tumor

## Abstract

Large cell neuroendocrine carcinoma, a type of non-small cell lung cancer, is quite rare and has been associated with brain metastasis, mainly to the cerebral hemispheres. However, the rate of cerebellar metastasis is underreported in the literature and appears to be quite rare. Despite the rarity of this metastasis, treatment guidelines for both supratentorial and cerebellar lesions have been established by using either radiosurgery or whole-brain radiation therapy. The choice of modality must take into consideration the vicinity of relevant structures such as the brainstem and its multiple nuclei. Here we report the case of a 68-year-old male, resident of a rural community in the Andean region of Ecuador, a low-middle income country; with the diagnosis of a large cell neuroendocrine carcinoma of the lung with dual central nervous system metastasis treated with linear particle accelerator radio-surgery due to its versatility and cost-effectiveness in a resource-limited setting. We showcase the rarity of the metastatic lesions as well as the utility of linear accelerators and their versatility to perform precise radiosurgical procedures in two simultaneous locations.

## Introduction

Large cell neuroendocrine carcinoma (LCNEC), a type of non-small cell lung carcinoma (NSCLC), is a rare type of cancer with a low but rising incidence of 3% [1]. It is estimated that 20% to 30% of patients with NSCLC will develop brain metastasis at some point in the natural progression of their disease [2]. Furthermore, brain metastasis occurs most commonly from primary lung cancers (two-third of brain metastasis, around 66%, come from a primary lung tumor) [3-5]. As of today, there is no clear management for both the primary lesions and metastases of LCNEC due to the limited number of cases, so most of the guidelines for the treatment of the disease are derived from those for SCLC or other NSCLC [6]. 

Thanks to radiosurgery with its different modalities (single fraction, multiple fractions, and adaptative) as well as the different devices used to administer the radiation (Gamma knife, LINAC, CyberKnife, Tomotherapy, etc.) the therapeutic spectrum of cerebral metastases from a primary pulmonary neuroendocrine tumor has broadened. This therapeutic approach has proven to have a better global survival rate compared to total cerebral radiation as well as a lower incidence of cognitive deterioration as a side effect of the treatment [7]. For example, Gamma knife has been extensively studied and multiple management guidelines have been produced for this specific patient group [8]. Nonetheless, due to the limitations of Gamma knife to perform other techniques or provide treatment in other locations, in low-to-middle income countries (LMICs), the versatility of use of LINACS with radiosurgery capabilities could be useful. Certainly, LINAC and Gamma knife are equivalent therapeutic techniques; however, in a place with limited resources, the acquisition of equipment that is able to administer different modalities of radiation therapy such as intensity-modulated radiation therapy (IMRT), volumetric modulated arc therapy (VMAT), image-guided radiation therapy (IGRT), and conformational radiotherapy, as well as being able to administer such treatment in different body regions, organs and tumors, is a cost-effective solution.

Here we present the case of a high-grade neuroendocrine carcinoma of the lung, stage III NSCLC, in a 68-year-old male patient from the parish of Aloag, province of Pichincha, Ecuador. Despite the lung tumor having adequate management, the patient developed brain and cerebellar metastases. These were initially managed through whole-brain radiotherapy (WBRT) due to the significant technological limitations of the first healthcare institution that the patient had access to and due to accessibility issues caused by the pandemic in all the radiosurgical services of the country. The definite treatment for the metastases was undertaken in a specialized cancer center through radiosurgery performed by LINAC, which is an equivalent alternative of treatment for multiple metastases when compared to other modalities such as Cyberknife or Gamma knife. Despite the limited access to therapeutic options in the country and thanks to the versatility of the LINAC machine, the patient was able to get efficient treatment even when the COVID-19 pandemic was causing difficulties in ensuring proper scheduling of therapeutic sessions; he remains asymptomatic to this day.

## Case presentation

The patient is a 68-year-old retired male, born in Aloag and resident of Tambillo (a rural locality in the vicinity of the capital of Ecuador, Quito). His medical history was significant only for being a heavy smoker until 2016 (with a calculated 20 pack-year), copious alcohol consumption every 15 days until 2010 and a myocardial infarction in 2015, successfully treated with stenting, acetylsalicylic acid and atorvastatin, a medication that he continues until this day. There is no family history of cancer or other pathologies of interest.

In February 2020, he presented dysesthesias in the right hemithorax associated with pain and a mass-like sensation in the same region. This prompted a visit to his local healthcare center (part of the public health network) where a chest CT scan was ordered in March 2020 revealing a solitary pulmonary mass located in the right inferior lobule with an invasion of both the pleura and thoracic wall. However, due to the beginning of the COVID-19 pandemic in Ecuador, all further studies were suspended for two to three months, resulting in a significant delay of the biopsy, which was undertaken on May 17, 2020. The histopathological study reported a neuroendocrine carcinoma. The patient was subjected to a thoracotomy and inferior pulmonary lobectomy on June 7, 2020 and was afterward treated with four cycles of chemotherapy consisting of cisplatin and etoposide until November of the same year. In December, the patient presents with neurologic symptoms consisting of loss of balance, ataxic gait, headaches, and nausea, prompting the necessity of a brain MRI. The study revealed a mass on the right lobe of the cerebellum (2.66 x 2.61 x 2.48cm) with perilesional edema, compressing the fourth ventricle. A progression of his primary lung cancer was diagnosed, the original chemotherapy regimen was suspended and replaced with adjuvant Temozolomide maintenance therapy, and he is referred to the radiotherapy service for WBRT with 20 Gy divided into five fractions, which improved his symptoms. 

**Figure 1:**
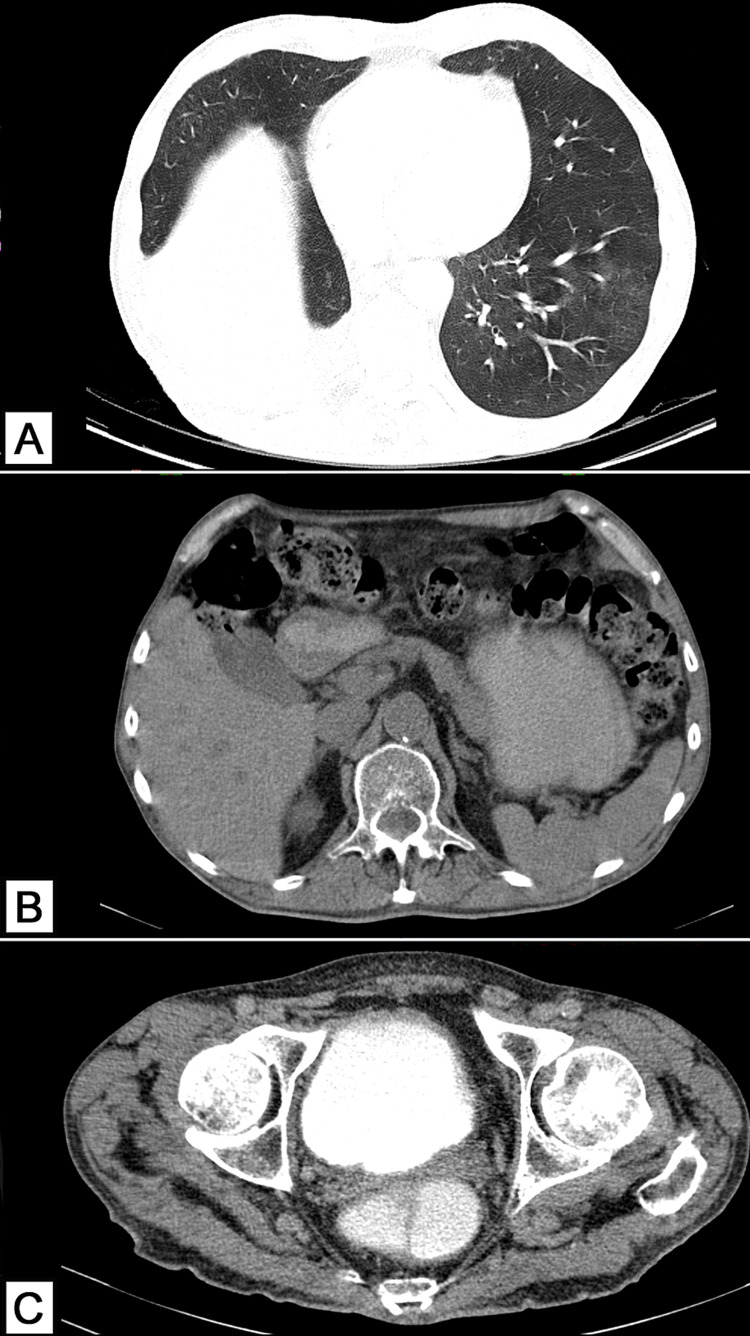
CT scan for recurrence and metastases detection (A) Axial CT scan (pulmonary window) is compatible with the patient’s history of right inferior pulmonary lobectomy; additionally, no masses or nodules are detected on either lung. (B) Normal abdominal CT scan with no signs of metastasis. (C) Normal pelvic CT scan with no signs of metastasis.

**Figure 2:**
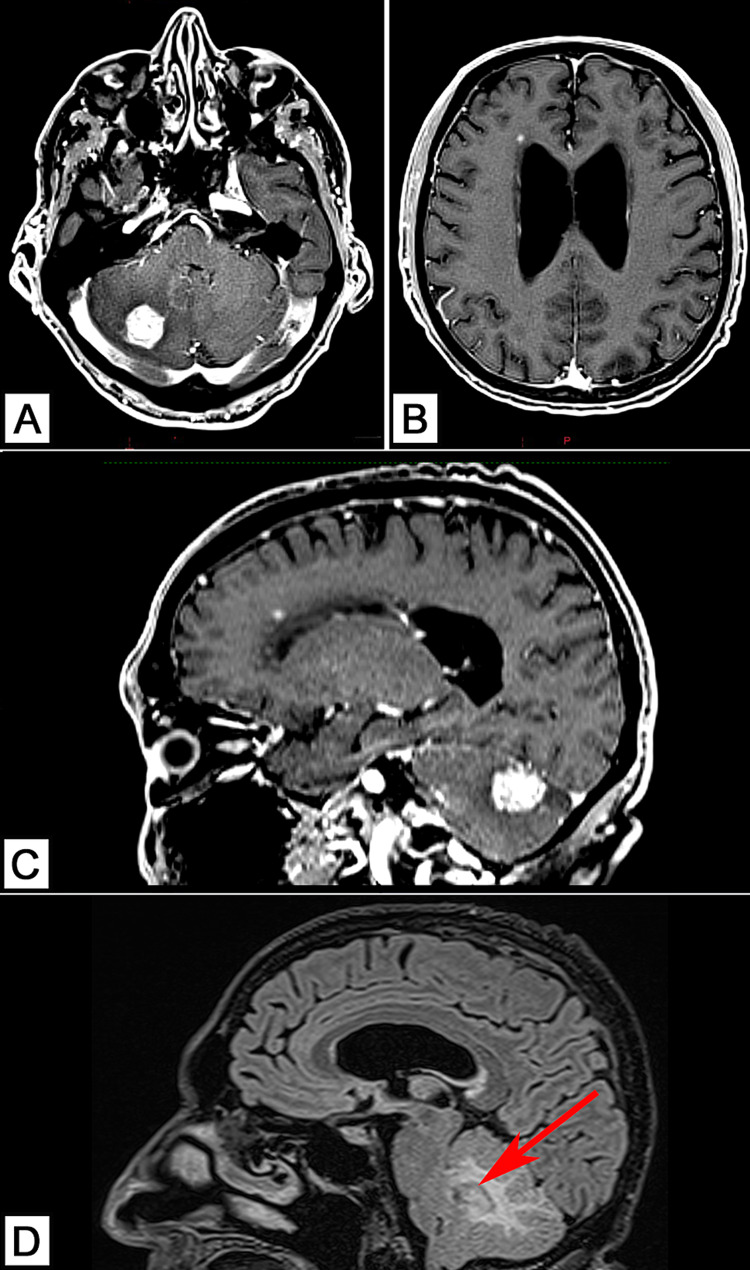
Pre-treatment brain MRI (A) Homogenous contrast-enhancing lesion localized in the right cerebellar hemisphere and causing significant vasogenic edema with mass effect, occluding the fourth ventricle. (B) Small homogenous contrast-enhancing lesion located anterior to the frontal horn of the right lateral ventricle, without any significant signs of edema. (C) Sagittal contrasted MRI showcasing the location of both lesions in the right cerebral and cerebellar hemispheres. (D) Sagittal FLAIR MRI revealing vasogenic edema and occlusion of the fourth ventricle (red arrow).

However, in March 2021, the patient comes to our hospital’s radiotherapy department to request a second opinion due to the reappearance of his neurological symptoms (loss of balance, headaches, nausea, dizziness, and photophobia). The physical examination is relevant for a Glasgow Coma Scale of 15, oriented in time, place, and person, incapacitating ataxic gait (the patient needs to be mobilized in a wheelchair), and severe photophobia requiring the constant use of sunglasses. The rest of the neurological examination is normal including cranial nerves, reflexes, and somatosensory function. Relevant prognostic indexes were calculated and included the Karnofsky Performance Scale (KPS) of 80%, and the Graded Prognostic Assessment (GPA), specific for patients with brain metastasis, of 3. A CT scan of the thorax, abdomen and pelvis was obtained, on February 22, 2021, in order to detect any new tumoral or metastatic activity; however, none was found (Figures 1A-1C). Therefore, a follow-up MRI was ordered on February 23, 2021, which revealed two intra-axial lesions on the right cerebellar hemisphere and near the right lateral ventricle, respectively (Figures 2A-2D).

**Figure 3:**
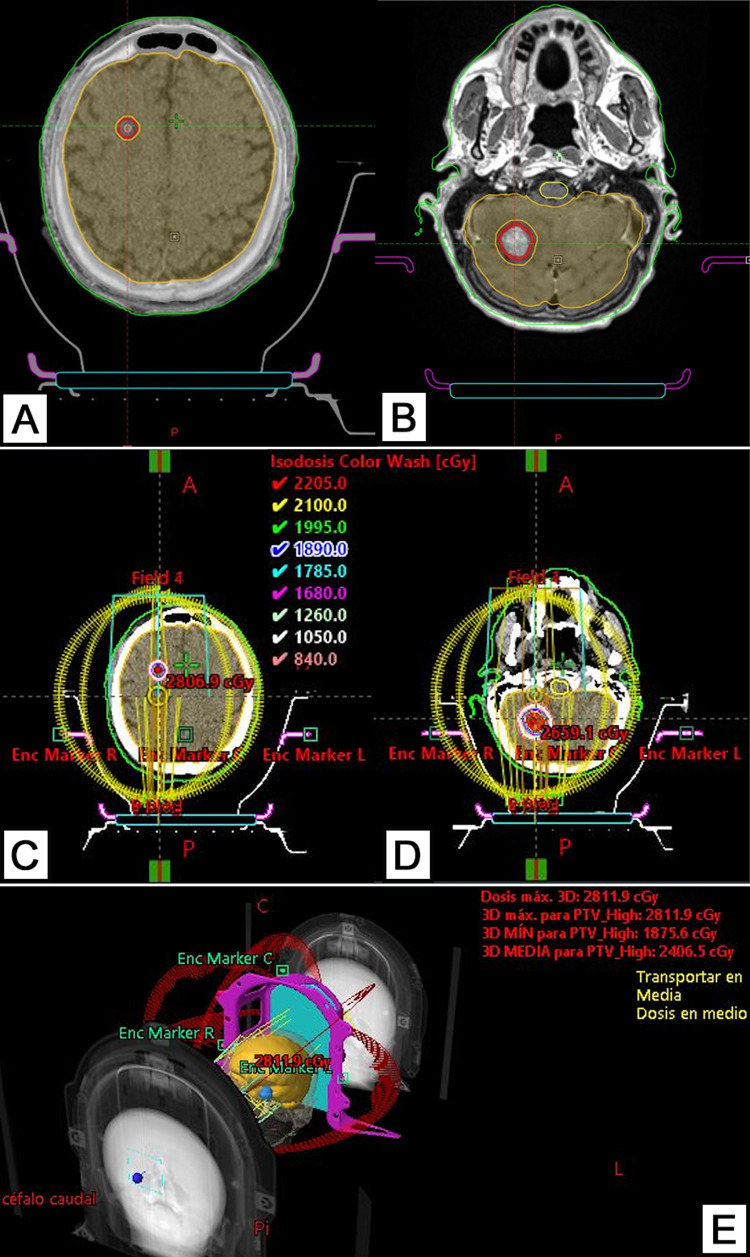
TrueBeam radiosurgery planning (A, B) The clinical planning of the cerebral and cerebellar lesions, respectively. (C, D) The dosimetric planning of the cerebral and cerebellar lesions, respectively. (E) Three-dimensional reconstruction of the radiosurgical planning and the trajectory of the beams.

Due to the aforementioned clinical and imaging findings, radio-surgical management of the disease with 21Gy, in one fraction, delivered with a linear particle accelerator (LINAC), TrueBeam® (Varian Medical Systems, Inc., Palo Alto, CA) was offered to the patient; immobilization was performed with a thermoplastic mask and Precise Bite mouthpiece (Solstice™ SRS Immobilization System, Orange City, IA); treatment simulation was done on the CT images and volume definition was performed in the contrasted T1 MRI with SPACE sequence; Gross tumor volume (GTV) was defined as the signal enhancing hyperintense tumoral regions observed on MRI and planning target volume (PTV) was prolonged 1mm in all directions. Dosimetric planning was based on HyperArc (Varian Medical System) which is a relatively new isocentric VMAT technique developed specifically for non-coplanar, multileaf collimator (MLC)-based stereotactic radiotherapy with automated treatment optimization and dose delivery; the clinical and dosimetric planning can be found in Figures 3A-3E. Dosage and organ constraints, evaluation of the iso-dosage curve goals as well as the evaluation of the Paddick index were performed in accordance with published guidelines [9,10]. Before treatment, we administer a 16mg intramuscular deposit dexamethasone injection in order to reduce the risk of cerebellar tonsil herniation due to the important edema from the cerebellar metastasis. Tomographic images obtained with the Cone Beam CT system, integrated into the LINAC, showed a complete (100%) correlation of the images obtained during simulation and planning. Finally, the effective time of treatment was of four minutes.

**Figure 4:**
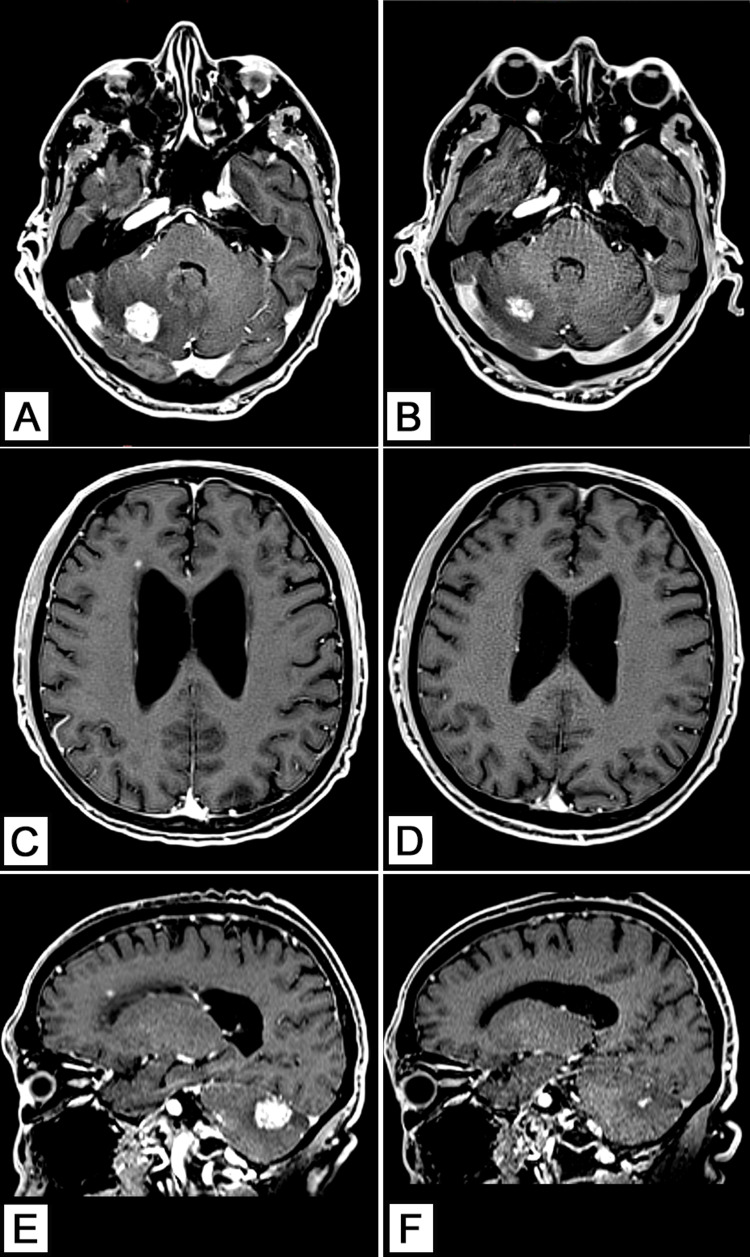
Pre- and post-treatment comparison scans (A, C, E) Pre-treatment contrast-enhanced MRIs. (B, D, F) Post-treatment scans showcasing a significant reduction in lesion size. (B) Post-treatment contrast-enhanced axial MRI shows a significant reduction of the lesion diameter and complete resolution of the mass effect affecting the fourth ventricle. (D) Post-treatment contrast-enhanced axial MRI reveals a complete resolution of the small metastatic lesion anterior to the frontal horn of the right lateral ventricle. (F) Post-treatment contrast-enhanced sagittal MRI clearly shows a remarkable reduction in the size of both lesions.

Treatment was delayed as per the patient’s request for more time to consider and on April 5, 2021, the procedure was finally performed without complications and in an outpatient setting. A follow-up was programmed 15 days after treatment at which the patient presented significant clinical improvement (absence of headaches, nausea, dizziness, photophobia, and normal balance and gait). There was no evidence of radiation toxicity, and the cognitive function was normal. A control MRI on July 17, 2021, showed a clear reduction in the size of both lesions as well as complete resolution of the mass effect produced by the cerebellar mass; a comparison of the pre- and post-treatment scans can be found in Figures 4A-4F.

At the moment, the patient remains in continuous follow-up, with the last reported telephonic follow-up, on September 2, 2021, in which the patient reported being asymptomatic and carrying out all of his regular day-to-day activities without any limitations.

## Discussion

LCNEC is rare cancer with a reported rate of 3% and an overall survival which varies from 8.7-10.2 months or of 80% at the first year, 41% at two years, and 17% at three years [1,2,4,11]. It is prevalent among older (>60 years) male patients with a history of smoking [12]. The treatment for localized disease follows partly the guidelines of treatment for SCLC and NSCLC consisting in surgical removal of the localized tumor plus neoadjuvant or adjuvant chemotherapy composed of a combined scheme of cisplatin and etoposide from four to six cycles [6,13-16]. Our patient’s clinical history is coherent with the reported age of presentation although his clinical debut deviates from what is reported in the literature since he did not present most of the symptoms commonly associated with an LCNEC of the lung (cough, hemoptysis, dyspnea, and night sweats); although, he did present an asymptomatic nodule and chest pain [13]. As mentioned earlier, through the biopsy the definite diagnosis of a high-grade stage 3 NSCLC was made, and the treatment mentioned beforehand was initiated.

In various studies, the sensitivity to platinum-based chemotherapy has been reported to be lower than that of SCLC, with approximately 43.8%-60% efficacy [6,11,12]. Due to this, there is a 30% recurrence after the excision of the primary tumor [2]. The brain is the first site of relapse in 25% (15%-40%) of localized high-grade stage III NSCLC [6], and LCNEC brain metastases appear in 25%-32.2% of the cases [15,17]. These metachronous brain metastases are associated with a better prognosis [18]. The time between diagnosis of the primary tumor and appearance of metachronous brain metastasis varies among different studies; it is reported to occur between 4.7 and 12.8 months on average [4,19]. The overall survival for NSCLC with metachronous brain metastases is reported to be 50% at one year, 30% at two years, and 20% at three years [2]. Our patient presented a relapse one month after the resection of the primary tumor, which is early compared to what is reported in the literature. Furthermore, there is scarce evidence available regarding the rate of LCNEC metastasis to the cerebellum, specifically. To the best of our knowledge, only one more case report can be found in the literature where a pulmonary LCNEC metastasizes to the cerebellum [20]. As of the writing of this case report, our patient remains disease-free and in a good clinical condition.

The treatment for brain metastases consists of WBRT or stereotactic radiosurgery (SRS) [7,16,21]. It has been described that SRS is used generally for a maximum of two to three metastatic lesions, with a maximum linear size of approximately 2.4cm (0.64-4.83cm), and a median lesion volume of 4.2cm^3^ (0.08-27.2cm^3^); this treatment modality has better local control and reduced rates of cognitive neurotoxicity [19]. WBRT is generally used when multiple metastases are present but has the downside of causing potentially irreversible cognitive impairment amongst other potential side effects such as patient-reported fatigue, short- and long-term memory changes, attention deficits, and depression [19]. On the initial intervention at the first point of care, WBRT was selected for the management of the metastasis followed by chemotherapy with temozolomide due to its ability to cross the blood-brain barrier based on the guidelines of NCCN for management of SCLC, which recommends a single chemotherapeutic agent as adjuvant treatment [12]. Despite the treatment installed, our patient relapsed after five months and was referred to our specialized cancer center for further treatment.

The use of SRS for the management of CNS metastasis of LCNEC has gained traction over recent years. Through the use of Gamma Knife authors such as Kotecha et al. and Kawabe et al. have been successful in treating metastatic brain lesions, yielding studies where they report cumulative incidences of local relapse of 13.8% in 12 months reported by Kawabe and 25% in 12 months reported by Kotecha [8,19]. Kotecha also reports 20.8 months of median survival after the use of SRS alone for the resolution of brain metastases [19]. Both authors recommend close monitoring and follow-up due to the risk of subclinical micrometastatic disease, which may lead to early relapse; however, it appears that SRS might be useful as a salvage procedure for new lesions and suffices in most cases [8]. In our report, we present the use of LINAC for the treatment of the cerebellar metastasis after the initial WBRT, which in the setting of an LMIC and performed in a specialized center, with properly trained personnel, can be used instead of Gamma Knife due to its versatility for performing radiotherapy and radiosurgery in multiple systems, which makes it a more cost-effective inversion for places with limited resources.

## Conclusions

The main highlights of this case report are that: 1) this appears to be the second case reported in the literature of an LCNEC metastasis localized in the cerebellum. 2) It serves as a precedent for the use of LINAC as an effective treatment for brain metastasis and indicates it is a useful tool particularly in LMICs due to the limited availability of resources. 3) The patient continues asymptomatic, 17 months after diagnosis, which doubles the time reported in the literature for overall survival. However, more studies of rigorous methodology are needed in order to properly ascertain the benefit and the indications of use of LINAC in NSCLC metastasis to the central nervous system.
